# Temporal Trends of Secondhand Smoke Exposure: Nonsmoking Workers in the United States (NHANES 2001–2010)

**DOI:** 10.1289/EHP165

**Published:** 2016-05-10

**Authors:** Binnian Wei, John T. Bernert, Benjamin C. Blount, Connie S. Sosnoff, Lanqing Wang, Patricia Richter, James L. Pirkle

**Affiliations:** Division of Laboratory Sciences, National Center for Environmental Health, Centers for Disease Control and Prevention, Atlanta, Georgia, USA

## Abstract

**Background:**

The workplace is one of the major locations outside of the home for nonsmokers’ exposure to secondhand smoke (SHS). New policies in many U.S. states and localities restrict or prohibit smoking in the workplace, and information on current trends in the exposure of nonsmokers to SHS across various occupational groups is therefore needed.

**Objective:**

We evaluated temporal trends in SHS exposure among nonsmoking workers in the United States and identified those occupations with workers with the highest levels of SHS exposure.

**Methods:**

We combined serum cotinine (sCOT) measurements and questionnaire data from five survey cycles of the National Health and Nutrition Examination Survey (NHANES: 2001–2010). Trends in SHS exposure by occupations were determined from percent changes and least-squares geometric means (LSGMs) of sCOT concentrations computed using sample-weighted multiple regression models.

**Results:**

Between NHANES 2001–2002 and NHANES 2009–2010, LSGMs of sCOT levels had changed –25% (95% CI: –39, –7%) in nonsmoking workers. The largest decrease was identified among food preparation workers [–54% (95% CI: –74, –19%)], followed by white-collar [–40%, (95% CI: –56, –19%)] and blue-collar workers (–32%, 95% CI: –51, –5%). LSGMs of sCOT remained highest in food preparation workers in all survey cycles, but the gap between occupations narrowed in the latest survey cycle (2009–2010). For example, the gap in LSGMs of sCOT between food preparation and science/education workers dropped > 70% during 2000 to 2010.

**Conclusions:**

During the period from 2001 to 2010, the overall SHS exposure in nonsmoking workers declined with substantial drops in food preparation/service and blue-collar workers. Although disparities persist in SHS exposure, the gaps among occupations have narrowed.

**Citation:**

Wei B, Bernert JT, Blount BC, Sosnoff CS, Wang L, Richter P, Pirkle JL. 2016. Temporal trends of secondhand smoke exposure: nonsmoking workers in the United States (NHANES 2001–2010). Environ Health Perspect 124:1568–1574; http://dx.doi.org/10.1289/EHP165

## Introduction

Second-hand smoke (SHS), that is, exposure of nonsmokers to tobacco smoke, has been shown to cause cancer and respiratory and cardiovascular diseases in nonsmoking adults and to cause serious respiratory problems in children [Schönherr 1928; Centers for Disease Control and Prevention (CDC) 2013; Department of Health and Human Services (DHHS) 2014]. The International Agency for Research on Cancer (IARC) and the National Toxicology Program (NTP) of the National Institutes of Health have classified SHS as a human carcinogen (IARC 2004; NTP 2014). In 2004, SHS reportedly caused > 600,000 deaths worldwide (Öberg et al. 2011). In the United States, despite the increasing awareness of adverse impacts of SHS exposure, and despite the implementation of smoke-free policies in many states, SHS remains a frequent air pollutant and a major preventable cause of premature deaths and disability. According to the 2014 Report of the Surgeon General, 41,000 estimated deaths per year are attributable to SHS (DHHS 2014).

Over the past two decades, many studies have addressed exposure to SHS within diverse settings. Hammond and colleagues measured air nicotine concentrations to examine tobacco smoke exposure in offices and production areas, and they found that SHS exposure posed a substantial risk to workers at worksites without smoking restrictions (Hammond et al. 1995; Hammond 1999). Understanding of the extent of SHS exposure within the U.S. general population improved when serum cotinine (sCOT), a metabolite of nicotine present in tobacco and tobacco smoke (Hukkanen et al. 2005), was measured in all participants ≥ 4 years old beginning with the Third National Health and Nutrition Examination Survey (NHANES III). Based on cotinine data collected in NHANES III, Pirkle et al. (1996) reported, for the first time, the extent of SHS exposure and differences among population groups within the general U.S. population. Subsequently, Wortley et al. (2002) reported variations in SHS exposure across different occupations. These studies revealed that disparities exist in SHS exposure levels across population groups in different environmental settings: that is to say, home and the workplace (Pirkle et al. 1996).

As of 30 June 2014, 26 states and Washington, DC have established comprehensive smoke-free indoor air laws for bars, restaurants, and worksites (CDC 2015), whereas there were few such policies before 1980. Several major events influencing smoking and health issues have also occurred over the past two decades, such as the availability of nicotine medications in 1996 (CDC 1997), the tobacco Master Settlement Agreement in 1998 (CDC 2014a), and the Family Smoking Prevention and Tobacco Control Act in 2009 (FDA 2009). Implementation of comprehensive smoke-free policies at state and local levels accompanied by national events such as those mentioned above could lead to a decline in SHS exposures in the United States.

In the present study, we combined and examined the sCOT concentrations and associated questionnaire data regarding occupation, tobacco use, and exposure to SHS collected in five consecutive cycles from the NHANES, conducted by the National Center for Health Statistics (NCHS) within the CDC, during 2001–2010. We evaluated SHS exposure among nonsmoking workers (≥ 16 years) with no reported smoker(s) at home across a wide range of occupational categories. The findings from this study establish SHS exposure levels among U.S. nonsmoking workers during 2000–2010 for comparison with future evaluations.

## Methods

### Study Design and Participants

The NHANESs are a series of cross-sectional health examination surveys representative of the U.S. civilian noninstitutionalized population that are conducted by the NCHS, CDC. The representative samples of participants are obtained through a complex, stratified, multistage probability design with unequal probabilities of selection. Written informed consent was obtained from all participants, and the protocol was approved by the NCHS Research Ethics Review Board.

NHANES data are released in 2-year cycles. The data included in this study for SHS exposure evaluation were from five consecutive survey cycles: 2001–2002, 2003–2004, 2005–2006, 2007–2008 and 2009–2010. We merged the survey data and calculated new sample weights for each participant according to NCHS recommendations (CDC 2014b). We restricted our analyses to participants ≥ 16 years old whose occupations were available. Sample sizes and characteristics for demographic and socioeconomic covariates are given in Table 1.

**Table 1:** Sample size characteristics of all nonsmoking workers in combined data set (NHANES 2001–2010).

NHANES 2001–2010^*a*^	Sample size	Unweighted percent, %
All	9,568	100.0
Sex
Male	4,690	49.0
Female	4,878	51.0
Age (years)
16–19	938	9.80
20–59	7,245	75.7
≥ 60	1,385	14.5
Race/ethnicity
Non-Hispanic white	4,280	44.7
Non-Hispanic black	1,802	18.8
Mexican American	2,264	23.7
Other	1,222	12.8
Ratio of family income to poverty (PIR)
PIR < 1.0	1,669	17.5
1.0 ≤ PIR < 2.0	1,886	19.7
2.0 ≤ PIR < 3.0	1,410	14.7
PIR ≥ 3.0	4,603	48.1
Education
Below high school	1,689	17.6
High school/ general educational development	1,718	18.0
Some college or associate’s degree	2,592	27.1
College graduate or higher	2,626	27.4
Not reported	943	9.90
^***a***^The combined data set was from five consecutive survey cycles: 2001–2002, 2003–2004, 2005–2006, 2007–2008 and 2009–2010.		

For comparison with the sample-weighted sCOT concentrations reported in the present study, we cited current cigarette smoking prevalence (defined as having smoked ≥ 100 cigarettes during the participant’s lifetime and currently smoking every day or some days) among working adults in the United States (2004–2010) from a study based on the National Health Interview Survey (NHIS) (CDC 2011a). Current working adults were defined as those who worked at their main paid job within the last week during the week before the interview.

### Laboratory Measurements

Blood samples were shipped to the CDC’s National Center for Environmental Health laboratory on dry ice from the collection site, and the serum samples were produced and stored at temperatures < –60°C until analysis. We analyzed sCOT using high-performance liquid chromatography (HPLC) coupled with atmospheric pressure ionization (API) tandem mass spectrometry (MS/MS) (Bernert et al. 2000; Bernert 2008). Approximately 5.95% of serum samples from nonsmoking workers in NHANES 2001–2002 were analyzed with a limit of detection (LOD) of 0.05 ng/mL, and the subsequent NHANES 2003–2010 samples were analyzed using an improved method with an LOD of 0.015 ng/mL. The overall intra- and interday accuracy and imprecision were < 10%. A previous study reported that little difference in statistical estimates as the “dilution” effect attributable to LOD was approximately comparable among different categories (Pirkle et al. 2006). A measured value at or above the LOD was classified as “detected” in our analyses. Calibration standards, quality control samples, and laboratory blanks were included in each analytical batch along with the study samples. Instruments were regularly evaluated to maintain the high sensitivity and reliability of the data. All reported biomarker results met the accuracy and precision specifications of the rigorous quality control/quality assurance program of the Division of Laboratory Sciences, National Center for Environmental Health, CDC (CDC 2014c).

### Statistical Methods

Statistical analyses were performed using SAS® software (version 9.3; SAS Institute Inc., Cary, NC, USA) and SUDAAN® (version 11.0.0; RTI International, Cary, NC, USA). We first merged the data regarding sCOT concentrations, tobacco, and occupation-associated questionnaire data, and then we calculated new sample weights for each participant according to the recommendations of the NCHS; these weights were equal to 1/5 of the 2-year sample weights provided in the demographic files (CDC 2014c). In all of our analyses, statistics were adjusted for the new sampling weights and for unequal selection probabilities and planned oversampling of certain subgroups resulting from the complex multistage probability design of NHANES.

We defined participants as nonsmokers if they had measured sCOT ≤ 10 ng/mL (Pirkle et al. 1996, 2006) and neither self-reported use of cigarettes nor of any other tobacco products, including cigar, pipe, snuff, chewing tobacco, nicotine patch, or gum, within the last 5 days before the survey. For adolescents 16–19 years old, those who self-reported smoking cigarettes within the preceding 30 days were also excluded. In all calculations, nonsmokers who lived with someone who smoked inside the home were excluded based on their response to the question, “Does anyone smoke inside your home?,” as were those who reported the use of any product containing nicotine to help stop smoking based on their response to the question, “Last time used nicotine stop-smoking aid.”

Occupations were categorized on the basis of the participant’s current job, which was defined as the main paid job worked within the last week. Occupation codes were based on the 2000 version of the U.S. Census Bureau codes in NHANES 2001–2006 (CDC 2005), and those during 2007–2010 were based on the 2002 version (CDC 2007). To ensure sufficient sample size for each occupation, we included workers even when they had worked < 35 hr per week based on their response to the question “Usually work 35 or more hours per week?”

To assess the relationship between sCOT concentration and NHANES release cycle, we conducted temporal trend analyses using sample-weighted multiple linear regression models. We first constructed a “core” linear regression model wherein the dependent variable was natural log–transformed sCOT concentration, the independent variable was the NHANES data release cycle, and the data collected in 2001–2002 served as the reference group. Here, sCOT was natural log–transformed because of the skewed distributions (Pirkle et al. 1996; Wei et al. 2016a, 2016b). To examine whether associations between NHANES release cycle and sCOT concentration varied by occupation after adjustment for demographic and socioeconomic covariates (age, sex, race/ethnicity, education, and household income) (Table 1 for categories), we added those covariates to the core regression model and then modeled multiplicative interactions between NHANES cycle and occupation by adding the product term to the model.

From the sample-weighted regression analysis, we estimated percent change in sCOT concentration of each occupation category by NHANES cycle as [exp(β) – 1] ×100% with 95% confidence intervals (CIs) estimated as [exp(upper/lower limits on β) – 1] ×100%, where β and upper/lower limits are the estimated regression coefficient and 95% CIs for β, respectively. We estimated least-squares geometric means (LSGMs) of sCOT concentration by release cycle as exp(least-squares means) with 95% CIs as exp(upper/lower limits on least-squares means), where the least-squares means are the cycle-specific means of sCOT concentration after adjustment for covariates. For concentrations below the LOD for sCOT, as recommended for analysis of NHANES data, the value of the LOD divided by the square root of 2 was used in the statistical analyses (CDC 2009). Statistics are presented only on measurements with sufficient frequency of detection (> 60%) to avoid undue influence on the estimates caused by imputed values in the analyses. In all analyses, a null hypothesis probability level of < 0.05 was considered to be statistically significant.

## Results

Among the five consecutive survey cycles (2001–2010), 9,568 respondents were identified as nonsmoking workers after excluding those who reported smokers in their home. The sample size characteristics of demographic and socioeconomic categories in the combined data set are given in Table 1.

Table 2 presents the seven occupation groups based on the similarities in current work types. Among them, sCOT was detected in 52.1–88.6% of all samples. Relative to the overall population of nonsmoking workers, sCOT was more frequently detected (> 80%) in workers who prepared and served foods. Nonsmoking workers in the science and education group generally had a lower detection rate for sCOT (52.1–69.8%) compared with other groups.

**Table 2:** Occupation category based on the similarity in current jobs of participants from NHANES 2001–2010.

Category	Occupation types
White-collar	Executives, administrators, and managers, and other management-related occupations.
Science and education	Engineering; architecture; computers; mathematical, life, physical, and social sciences; education; teaching; training; and library occupations.
Health-related	Health diagnosing, assessing, treating, related healthcare practitioner, technical support, and personal care and service.
Sales, finance, business-related	Sales supervisors and proprietors, sales representatives, finance, business, commodities, sales workers, retail, personal services, and other sales-related occupations.
Office, administrative support	Secretaries; stenographers; typists; information clerks; record processing, material recording, scheduling, and distributing clerks; miscellaneous administrative support workers.
Food preparation and service	Waiters and waitresses, cooks, and miscellaneous food preparers and servers.
Blue-collar	Workers performing cleaning and building services; vehicle and mobile equipment mechanics and repairers; other mechanics and repairers; workers in construction trades, extractive and precision production; textile, apparel, and furnishings machine operators; machine operators; assorted materials fabricators, assemblers, inspectors, and samplers; motor vehicle operators; other transportation and material movers; construction laborers; freight, stock, and material movers; other helpers; equipment cleaners; hand packagers and laborers.

**Figure 1:**
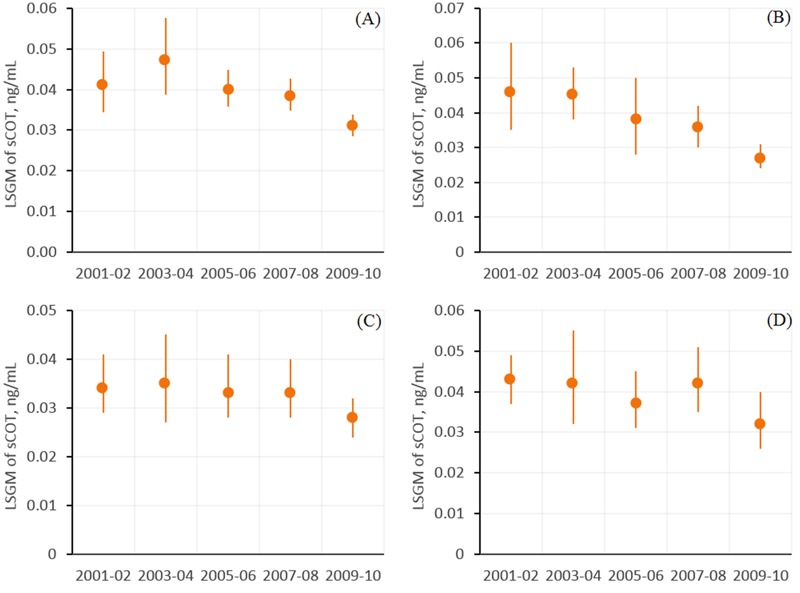
Association between serum cotinine (sCOT) concentrations (nanograms/milliliter) and NHANES release cycle for (*A*) all nonsmoking workers, (*B*) white collar workers, (*C*) science and education workers, and (*D*) workers in health-related occupations. Estimates in (*A*) are from regression models where the independent variable was NHANES release cycle, after adjustment for occupation, age, sex, race/ethnicity, education and PIR (ratio of family income to poverty). Estimates in (*B,C,D*) are from regression models of interactions between NHANES release cycles and occupation groups (Table 2), after adjustment for age, sex, race/ethnicity, education and PIR. Data points represent least-squares geometric means (LSGMs), and error bars indicate 95% confidence intervals. Corresponding numeric data are provided in Table 3.

**Figure 2:**
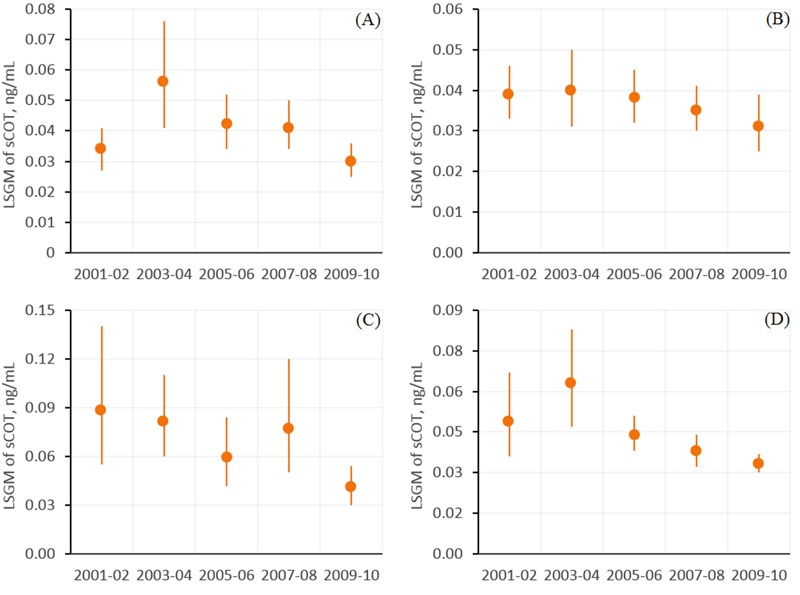
Association between serum cotinine (sCOT) concentrations (nanograms/milliliter) and NHANES release cycle by occupation for (*A*) workers in sales, finance, and business-related occupations, (*B*) office and administrative support workers, (*C*) food preparation and service workers, and (*D*) blue-collar workers. Estimates are from regression models of interactions between NHANES release cycles and occupation groups (Table 2) after adjustment for age, sex, race/ethnicity, education, and PIR (ratio of family income to poverty). Data points represent least-squares geometric means (LSGMs), and error bars indicate the 95% confidence intervals. Corresponding numeric data are provided in Table 3.

The LSGM of sCOT in all nonsmoking workers was significantly lower in 2009–2010 than in 2001–2002 (*p* = 0.008) after adjustment for occupation, age, sex, race/ethnicity, education, and household income (Figure 1A). Compared with 2001–2002, the LSGMs of sCOT were lower by 40% (95% CI: –56, –19%), 24% (95% CI: –42, –1%), 54% (95% CI: –74, –19%), and 32% (95% CI: –51, –5%) in 2009–2010 for nonsmoking workers categorized in white-collar (Figure 1B), health-related (Figure 1D), food preparation/service (Figure 2C), and blue-collar (Figure 2D) occupation groups, respectively (Figures 1 and 2, Table 3). During the same period from 2001 to 2010, LSGMs of sCOT changed –20% (95% CI: –37, 2%), –12% (95% CI: –33, 17%) and –20% (95% CI: –39, 6%) among nonsmoking workers categorized in science and education (Figure 1C), sales/finance/business-related (Figure 2A), and office administrative support (Figure 2B) occupation groups, respectively, but the differences in these percentage changes were not statistically significant.

**Table 3:** Association between serum cotinine concentrations (nanograms/milliliter) and NHANES release cycle by occupation category. Estimates were computed from sample-weighted linear regression model of interactions between release cycle and occupations, after adjustment for age, sex, race/ethnicity, education and PIR (ratio of family income to poverty).

Category/release cycle	LSGMs (95% CIs)	% Change	*p*-Value	Sample size	Detection rate, %
White-collar
2001–2002	0.046 (0.035, 0.060)	*Reference*	—	137	65.0
2003–2004	0.045 (0.038, 0.053)	–2 (–30, 35)	0.881	144	73.6
2005–2006	0.038 (0.028, 0.050)	–18 (–45, 24)	0.339	154	69.5
2007–2008	0.036 (0.030, 0.042)	–22 (–44, 8)	0.132	181	72.4
2009–2010	0.027 (0.024, 0.031)	–40 (–56, –19)	0.001	240	66.7
Science and education
2001–2002	0.034 (0.029, 0.041)	*Reference*	—	190	52.1
2003–2004	0.035 (0.027, 0.045)	2 (–25, 39)	0.890	129	69.8
2005–2006	0.033 (0.028, 0.041)	–2 (–25, 27)	0.848	221	69.7
2007–2008	0.033 (0.028, 0.040)	–3 (–25, 25)	0.810	216	62.0
2009–2010	0.028 (0.024, 0.032)	–20 (–37, 2)	0.068	257	60.3
Health-related
2001–2002	0.043 (0.037, 0.049)	*Reference*	—	176	67.6
2003–2004	0.042 (0.032, 0.055)	–1 (–28, 35)	0.949	168	72.0
2005–2006	0.037 (0.031, 0.045)	–12 (–30, 10)	0.261	219	74.4
2007–2008	0.042 (0.035, 0.051)	–1 (–23, 26)	0.906	224	79.5
2009–2010	0.032 (0.026, 0.040)	–24 (–42, –1)	0.045	250	66.0
Sales, finance, business-related
2001–2002	0.034 (0.027, 0.041)	*Reference*	—	288	64.2
2003–2004	0.056 (0.041, 0.076)	66 (13, 142)	0.010	243	81.5
2005–2006	0.042 (0.034, 0.052)	25 (–8, 68)	0.148	286	78.0
2007–2008	0.041 (0.034, 0.050)	23 (–8, 64)	0.168	293	75.4
2009–2010	0.030 (0.025, 0.036)	–12 (–33, 17)	0.385	297	68.0
Office, administrative support
2001–2002	0.039 (0.033, 0.046)	*Reference*	—	289	67.5
2003–2004	0.040 (0.031, 0.050)	2 (–24, 38)	0.876	252	74.2
2005–2006	0.038 (0.032, 0.045)	–2 (–23, 25)	0.870	273	70.7
2007–2008	0.035 (0.030, 0.041)	–10 (–29, 13)	0.360	263	72.6
2009–2010	0.031 (0.025, 0.039)	–20 (–39, 6)	0.125	238	66.4
Food preparation and service
2001–2002	0.088 (0.055, 0.140)	*Reference*	—	125	80.0
2003–2004	0.081 (0.060, 0.110)	–8 (–48, 63)	0.784	114	85.1
2005–2006	0.059 (0.042, 0.084)	–33 (–63, 21)	0.184	123	82.9
2007–2008	0.077 (0.050, 0.120)	–12 (–54, 69)	0.697	132	88.6
2009–2010	0.041 (0.030, 0.054)	–54 (–74, –19)	0.007	132	80.3
Blue-collar
2001–2002	0.049 (0.036, 0.067)	Reference	—	479	75.0
2003–2004	0.063 (0.047, 0.083)	28 (–16, 96)	0.250	389	83.8
2005–2006	0.044 (0.038, 0.051)	–10 (–36, 27)	0.547	511	86.5
2007–2008	0.038 (0.032, 0.044)	–23 (–46, 10)	0.146	551	80.8
2009–2010	0.033 (0.030, 0.037)	–32 (–51, –5)	0.026	632	77.2
Abbreviations: CI, confidence interval; LSGM, least-squares geometric mean.					

During the period 2000–2001, nonsmoking workers in the food preparation and service category had the highest LSGM of sCOT (0.088 ng/mL, 95% CI: 0.055, 0.140 ng/mL) among all groups, which was ~158% higher than workers in the science and education category (0.034 ng/mL, 95% CI: 0.029, 0.041 ng/mL). During the period 2009–2010, the difference in the LSGM of sCOT between those two groups decreased to 46%, suggesting that the SHS exposure gap narrowed over time.

**Figure 3:**
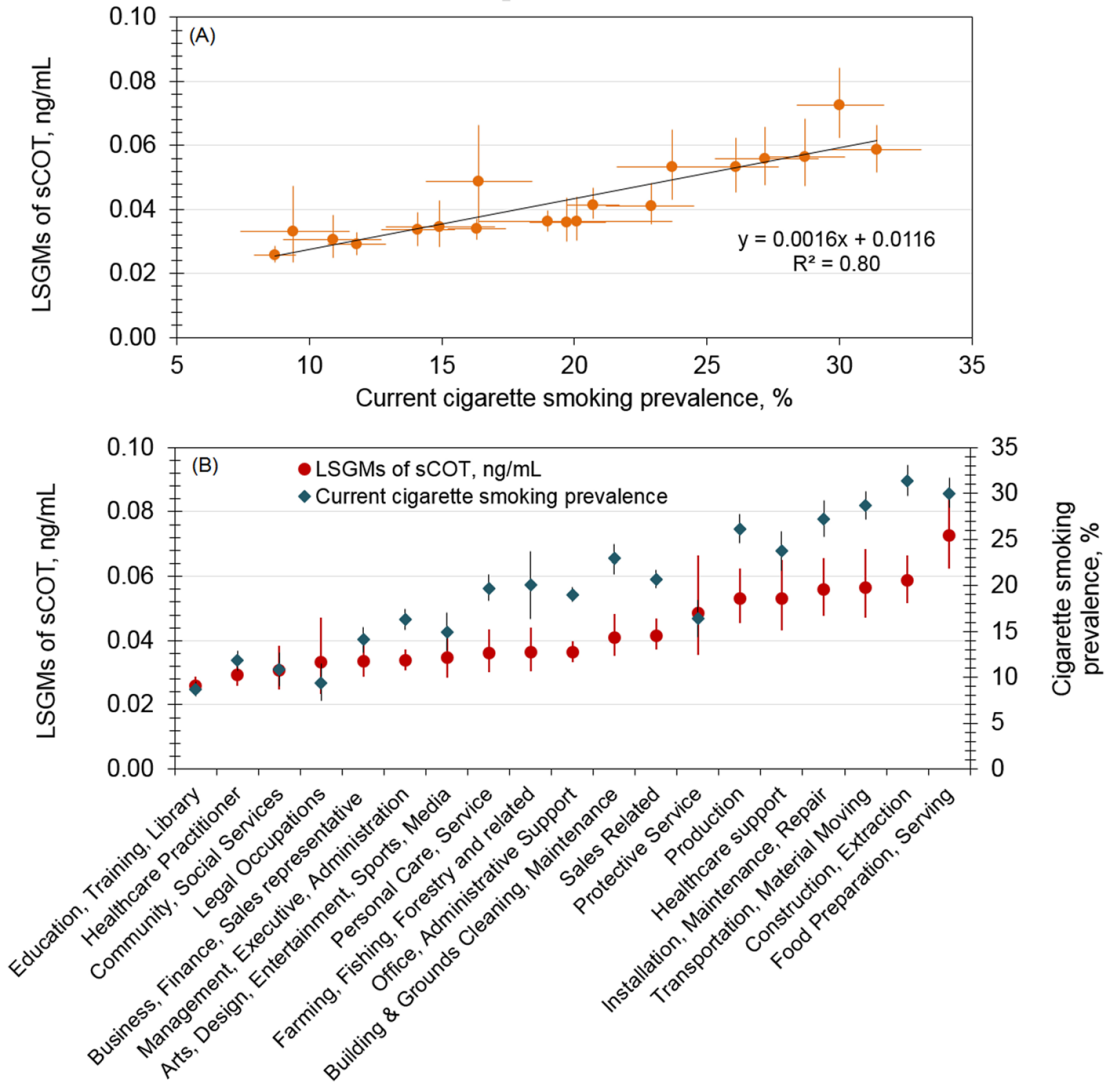
Serum cotinine levels among nonsmoking workers (NHANES 2001-2010) and cigarette smoking prevalence among working adults (NHIS 2004-2010). (*A*) Association by a simple regression model; (*B*) association by occupation category ranked according to serum cotinine level. Vertical and horizontal error bars indicate the 95% confidence intervals for sample-weighted geometric means of serum cotinine levels and current cigarette smoking prevalence, respectively. Current cigarette smokers are working adults who reported having smoked ≥ 100 cigarettes during their lifetime and currently smoking every day or some days.

Figure 3A,B shows the relationship between sample-weighted GMs of sCOT concentrations by occupation groups and by current cigarette smoking prevalence among working adults who reported having smoked ≥ 100 cigarettes during their lifetime and currently smoking every day or some days. Among the 20 detailed occupational categories, participants who worked in fields such as education, training, and libraries had the lowest GM of sCOT (0.026 ng/mL), followed by those working in the areas of science, technology, and engineering (0.028 ng/mL). Conversely, participants who prepared and served food had the overall highest GM of sCOT (0.077 ng/mL), followed by participants with construction and extraction jobs (0.060 ng/mL). These data also suggest an association between nonsmoking workers’ sCOT concentrations as an indicator of SHS exposure and the prevalence of smoking among adult workers. The overall data show a positive correlation between those two groups, with a squared correlation coefficient (*R^2^*) of 0.80.

## Discussion

Compared with the results from NHANES III (1988 to 1994) by Wortley et al. (2002), the overall sCOT in the 2001–2002 survey decreased approximately 78% among nonsmoking U.S. workers after those who self-reported home exposure to cigarette smoke were excluded. During the period from 2001 to 2010, a further significant decrease in the LSGM of sCOT concentration was detected (–25%, 95% CI: –39, –7%). This finding is consistent with survey data showing an overall slightly declining trend of cigarette use prevalence among working adults during the same time period (CDC 2011a). We have noted that the largest decrease in sCOT occurred in the 2009–2010 survey. However, the available data did not allow us to evaluate the impact of specific influential factors on this decrease. Generally, these decreases may be attributable to tobacco control progress made during the period, including the increasing number of states (as well as Washington, DC) with comprehensive smoke-free laws that prohibit smoking in indoor areas of worksites, restaurants, and bars (CDC 2011b).

We observed differences in sample-weighted GMs of sCOT concentrations among some occupations. For example, the sCOT concentrations in workers in the food preparation and service sectors (0.041–0.088 ng/mL) were nearly twice those in workers in educational and science-related sectors (0.028–0.034 ng/mL). These findings are similar to those previously reported for the NHANES III (1988 to 1994) population (Wortley et al. 2002), in which workers in the food preparation and service sectors also had high mean sCOT concentrations (0.24–0.47 ng/mL), although the overall mean sCOT concentrations for nonsmoking workers were far higher during the period studied by Wortley et al. (2002), that is, the late 1980s and early 1990s, than we observed in the more recent surveys. Again, this difference most likely reflects substantial public health accomplishments in reducing SHS exposure over the past two decades. Although the present lower SHS exposures are encouraging, particularly the narrowing of gaps between occupations over the period from 2001 to 2010, consistent differences among groups remain. For example, construction, extraction, production, transportation, and material moving workers had the highest sample-weighted sCOT levels after food servers in our study, whereas education, training, and library sector workers had the lowest SHS exposures. Although farming, fishing, and forestry workers tended to have relatively lower sCOT concentrations than might be expected, both in our study and in the earlier report by Wortley et al. (2002), this finding might reflect the predominantly outdoor work settings for these categories.

These persistent disparities in SHS exposure for workers in certain occupations may reflect differences in the strength and impacts of smoke-free policies across different occupational sectors. For example, blue-collar and service workers continue to encounter workplaces without smoke-free policies, whereas comprehensive coverage percentages for other occupations, such as white-collar workers, are far higher [American Nonsmokers’ Rights Foundation (ANR) 2016]. The potential importance of such policies in reducing SHS exposures in the workplace is well known (King et al. 2014). These policies may be particularly important for food servers, whose exposure depends not only on coworkers’ behaviors in a nonrestricted workplace but also to an important extent on other important factors such as their unavoidable proximity to smoking customers.

As in the home, public areas, social settings, and so forth, workplace SHS exposure is affected by the behavior of smokers in the vicinity of nonsmoking workers, such as the number of cigarettes smoked and the smoking rates. Recent data indicate that blue-collar workers continue to have higher smoking rates than do other types of workers despite the overall decline in cigarette smoking rates among U.S. adults (Chin et al. 2012; Chiu et al. 2010; Plescia et al. 2005). Fujishiro et al. (2012) reported that blue-collar workers are more likely than white-collar workers to be heavy smokers. Thus, factors such as higher smoking rates and a “smoke-friendly atmosphere” in the workplace are likely to be important contributors to the higher SHS exposure levels observed among nonsmoking blue-collar workers.

Our results are also consistent with those of other national surveys. Based on NHIS data, the prevalence of current cigarette smoking was ~19.3% overall among adult U.S. workers in 2010, with substantial differences in smoking prevalence across occupation groups. However, workers in the construction, extraction, and food sectors had smoking prevalence rates ~50% greater than the mean (CDC 2011b). The exposure of nonsmokers in the workplace might be expected to approximately parallel the extent of smoking to which they are exposed, and our finding of relatively higher nonsmoker sCOT levels in these same groups is consistent with this expectation. Conversely, smoking prevalence was reportedly < 9% among workers in education, training, and library sectors in the NHIS survey; this finding is in good accord with the low serum cotinine GMs that we observed among nonsmoking workers in these categories, which were among the lowest identified in our study. These findings suggest that cigarette smoking prevalence is one of the most influential factors affecting SHS exposure levels among nonsmoking workers.

The goal of reduction and eventual elimination of SHS exposure in the workplace will require not only universal coverage of smoke-free policies but also strong policy enforcement. King et al. (2014) found significantly fewer reports of exposure to SHS in workplaces with smoke-free policies (16.4%) than in workplaces lacking such policies (51.3%), but their findings also indicated that enforcement of these restrictions is not always reliable. In a study on workplace SHS exposure in the U.S. trucking industry (Chiu et al. 2010), only 23% of nonsmokers and 10% of smokers reported that smoke-free policies were always enforced. In a study addressing SHS exposure among workers in North Carolina, Plescia et al. (2005) reported that 3% of workers had violated the company smoke-free policy. Even one person violating a smoke-free policy can result in multiple nonsmokers being exposed to SHS. Thus, the current limited comprehensive smoke-free policies at workplaces and the lapses in their enforcement continue to complicate the issue of SHS exposures of nonsmokers in the workplace and suggest that individual employers should strive for working environments that are 100% SHS-free to finally accomplish the goal of comprehensive smoke-free workplaces (Calvert et al. 2013; NIOSH 2014); furthermore, additional challenges must be overcome to eventually eliminate health risks from SHS exposure among U.S. workers.

Our findings should be interpreted in the context of several limitations. First, the sCOT concentrations reflect the integrated contributions from all potential exposure sources, including inhalation, dermal absorption, and ingestion (McGuffey et al. 2014; Wei et al. 2014), and can be further complicated by factors such as the activities and exposure durations in the different environments that the participants had visited each day. Although we excluded participants with self-reported smokers inside their homes, neither potentially important exposures to outside SHS that penetrated through doors and windows nor the possibility of SHS exposure from visits to the home by smokers could be ruled out. Second, at present, it is not feasible to differentiate workplace SHS exposure, using sCOT concentrations, from exposures obtained from other sources, such as public areas and social settings. Thus, this study only indirectly evaluated workplace SHS exposure by assuming that nonworkplace SHS exposures occurred at comparable levels across different occupations. Third, this study combined a sCOT cut point of ≤ 10 ng/mL with responses to tobacco use questions as the criteria to select nonsmokers. Benowitz et al. (2009) proposed an overall cut point of 3 ng/mL and race/ethnicity-specific cut points of 1–6 ng/mL based on NHANES data collected during 1999–2004. A cut point ≤ 10 ng/mL could exclude misclassified smokers from the nonsmoker group; however, this cut point may also exclude participants who were self-identified nonsmokers with heavy exposure to SHS. Fourth, the occupation of each participant was identified using the self-reported information on his/her current job. Because of a low response rate for the question of total hours worked per week, we included workers who reported < 35 working hours per week to ensure adequate sample sizes for each occupation. In addition, during the 2001–2006 survey period, occupation codes were based on the 2000 U.S. Census Bureau codes, whereas during the 2007–2010 survey period, occupation codes were based on the 2002 U.S. Census Bureau codes. For instance, during the early survey periods (2000–2004), workers were categorized in 41 occupational groups, while during the survey periods from 2005 to 2010, workers were coded in 23 occupational groups. Thus, variations could exist when we grouped subjects into similar work categories. Fifth, although the majority of samples were analyzed using the improved method (LOD of 0.015 ng/mL), the less sensitive method (LOD of 0.05 ng/mL) used for the 5.95% of samples collected from nonsmoking workers in the survey cycle from 2001 to 2002 could have led to lower detection rates in these samples and could have resulted in potential variations across the categories. Finally, although we excluded all participants who self-reported use of cigarettes or other tobacco products including cigars, pipes, snuff, chewing tobacco, patches, or gum, some participants who were light or occasional smokers and had serum cotinine ≤ 10 ng/mL could have been included if these participants were misclassified as nonsmokers based on their responses to tobacco use questions. Participants reporting occasional use of other tobacco-related products such as e-cigarettes and with serum cotinine levels ≤ 10 ng/mL may also have been included because questionnaire data to identify and exclude users of these tobacco products during the study period (2001–2010) were not available.

## Conclusion

Our analysis of serum cotinine concentrations among nonsmoking U.S. workers ≥ 16 years of age showed that their SHS exposure declined 25% during the period from 2001 to 2010. Despite this progress, the observed serum cotinine concentrations suggest that disparities in exposure to SHS persist for nonsmokers in certain worker groups (i.e., food preparation/service workers). Overall, the temporal trends suggest that the exposure gaps among occupational groups have narrowed in the last two decades.
